# Research methods at the intersection of gender diversity and autism: A scoping review

**DOI:** 10.1177/13623613241245595

**Published:** 2024-04-25

**Authors:** Em JE Mittertreiner, Elise Ng-Cordell, Alana J McVey, Connor M Kerns

**Affiliations:** 1 The University of British Columbia, Canada; 2 Department of Psychiatry and Behavioral Sciences, University of Washington, Seattle, WA, USA; 3 Autism Center, Seattle Children’s Hospital, Seattle, WA, United States

**Keywords:** autism, autism spectrum conditions, autism spectrum disorders, gender diversity, gender dysphoria, methodological quality assessment, research methods, scoping review, trans, transgender

## Abstract

Research has increasingly focused on the intersection between gender diversity and autism. Understanding the foci, methods, and rigor of recent studies could help guide and maximize impact in this accelerating research area. We conducted a scoping review of peer-reviewed literature on this topic published between 2018 and 2023. The search generated 1432 records after deduplication. Included studies (*N* = 84) were of English language, featured original qualitative or quantitative findings, and examined a psychosocial connection between autism and gender spectra variables. Autism prevalence among gender-diverse people was the most-studied sub-topic. Methodological rigor was acceptable overall; however, we identified recurrent threats to generalizability and validity, including inconsistent conceptualization of constructs (e.g. gender dysphoria), weak participant sampling and characterization, and reliance on unvalidated measures. Addressing these limitations and meaningfully engaging with community shareholders will be critical to enhancing the replicability and clinical impact of future research.

**Lay Abstract:**

Research has increasingly focused on the intersection between gender diversity and autism. To better understand this literature, this scoping review systematically searched five databases for peer-reviewed literature on gender diversity and autism published between 2018 and 2023. Included studies (*N* = 84) were of English language, featured original qualitative or quantitative findings, and examined a psychosocial connection between autism and gender spectra variables. Most studies focused on measuring prevalence of autism among gender-diverse individuals. While the overall study rigor was acceptable, weaknesses in measurement, sample selection, and definition of key terms were noted. Promisingly, studies in this area appear to be shifting away from a pathologizing lens and towards research methods that engage in meaningful collaboration with the autistic, gender-diverse community to investigate how to best enhance the quality of life and wellbeing of this population.

When interviewed by Strang, Powers, et al. (2018) about their experiences accessing healthcare services, one gender-diverse, autistic person commented:

So when I first came out they said something like, “you know there is a lot of overlap between people who are on the spectrum and people who are transgender.” I wondered if they meant, ‘Are you sure this isn’t just the autism talking?’ (p. 4049)

Social and cultural norms related to personal identity—such as gender, ability, ethnicity, and sexual orientation—influence society’s perception of “normal” behavior. People who deviate from norms often face oppression, including ostracization, discrimination, and psychopathologization. According to Crenshaw’s (1989) theory of intersectionality, people who deviate from multiple norms experience unique, compounding effects. As articulated earlier, this is often a reality for people whose identities lie at the intersection of non-normative gender identity (i.e. gender diversity) and autism.

Terminology use is evolving in both autism and gender diversity research. In this review, we use the term “autism” in lieu of autism spectrum disorder and identity-first language, “autistic individual” and “autistic characteristics,” for autism-related behaviors that vary in intensity across the population (Bottema-Beutel et al., 2020). *Gender diverse* is an umbrella term describing people who express themselves in ways incongruent with the societal norms expected of their *sex assigned at birth* (SAB). Gender-diverse people may use labels such as transgender/trans, nonbinary, genderqueer, agender, genderfluid, and many more. Some—but not all—exhibit *gender dysphoria* (GD; i.e. impairing distress about the incongruence between gender expression and SAB; American Psychiatric Association, 2013). There are several barriers (e.g. pervasive transphobia within the medical system) that prevent gender-dysphoric people from seeking medical care (Bauer et al., 2009). Thus, Arcelus and colleagues (2015) suggested the prevalence of GD is likely greater than the 0.0046% indicated by their meta-analysis. Prevalence estimates for transgender identity are also higher, as not all trans people experience GD (0.5%–1.3%; Zucker, 2017).

Research has pointed to potential associations between gender diversity–related constructs and autism. For example, Nabbijohn et al. (2019) found gender variance was associated with autistic traits among a mixed clinical and community sample of children. Hisle-Gorman et al. (2019) found autistic children were over four times more likely than allistic children to have a GD diagnosis. Chart review studies also suggest above-average rates of autism diagnoses among gender-dysphoric adults (Cheung et al., 2018; Fielding & Bass, 2018; Heylens et al., 2018). However, a series of reviews published in 2018 (Turban & van Schalkwyk, 2018; van Schalkwyk, 2018) highlighted several methodological challenges in studies published by that time, including inconsistent operational definitions, use of measures that had not been validated among gender-diverse or autistic samples, uncontrolled confounding factors (e.g. the socioemotional effects of gender discrimination), a lack of comparison samples, and small sample sizes, all of which hinder replicability and generalizability. The extent to which these issues persist is not well understood, although this body of literature has rapidly expanded since that time (Kallitsounaki & Williams, 2023). To ensure this growing interest translates to higher-quality empirical knowledge, a better understanding of the current research challenges is needed.

## Rationale

To assess the range and rigor of research on the intersection of autism and gender diversity *since* 2018, we conducted a scoping review, a method which aims to provide a high-level view of a field’s strengths and weaknesses by comprehensively mapping research approaches. To date, there are six relevant systematic or scoping reviews (Øien et al., 2018; Glidden et al., 2016; Herrmann et al., 2020; Manjra & Masic, 2022; Thrower et al., 2020; van der Miesen et al., 2016) and one systematic review and meta-analysis (Kallitsounaki & Williams, 2023). Importantly, the current study aims to address remaining knowledge gaps. First, existing reviews focused on synthesizing findings to compare rates of co-occurring autism/autistic traits and GD/gender diversity. The heterogeneity of study methodologies, however, makes it difficult to draw overarching conclusions and underscores the need for a scoping review that synthesizes and critiques study *methods*. Second, identified reviews analyzed studies published prior to October 2020, and study methodologies have likely been honed and developed since then—especially qualitative methodologies, which are not examined by most existing reviews. Third, except for Manjra and Masic (2022)—a narrative review focusing on quantitative youth studies—prior reviews did not conduct assessments of methodological quality. Finally, studies of additional factors at play in autistic, gender-diverse people’s lives (e.g. wellbeing, co-occurring problems, quality of life) have historically been excluded. To address these limitations and respond directly to researchers’ calls for systematic documentation of methodological rigor (e.g. Glidden et al., 2016; Herrmann et al., 2020), we conducted a synthesis and quality assessment of the methods used in recently published studies examining the overlap between gender diversity/GD and autistic characteristics/autism. Publication date was restricted to between January 2018 and July 2023, because several reviews have examined studies published prior to 2018. Unlike past reviews, this review includes quantitative and qualitative studies of both adults and youth, and focuses on assessing overall study quality rather than collating results.

## Aims

1. To record the foci, research designs, and sampled populations.
2. To analyze how studies defined key concepts and terms related to the gender and autism spectra.
3. To evaluate methodological strengths and weaknesses and recommend future directions.

By addressing these aims, this review uses a data-driven approach to systematically evaluate and refine concerns raised about the methodological rigor of a rapidly evolving literature.

## Methods

This project adhered to the Joanna Briggs Institute (JBI) guidelines for scoping reviews (Aromataris & Munn, 2020) and Preferred Reporting Items for Systematic Reviews and Meta-Analyses—Extension for Scoping Reviews (PRISMA-ScR; Tricco et al., 2018).

### Inclusion criteria

Each study met the following *a priori* criteria: (1) published in an English-language peer-reviewed journal after December 2017; (2) featured empirical qualitative or quantitative research; (3) involved humans older than 2 years; (4) incorporated clinical (e.g. diagnoses, treatments), psychological (e.g. traits, behaviors), or social (e.g. interpersonal interactions) variables related to the gender or autism spectra; and (5) studied the existence or strength of an association between GD/gender diversity and autism/autistic characteristics.

Studies focused on biological phenomena (i.e. SAB, chemistry, anatomy, genes) were outside the review scope. We also excluded literature and systematic/scoping reviews, commentaries, editorials, conference proceedings, dissertations, gray literature, and case studies with <3 participants.

### Search strategy

We conducted a preliminary search of MEDLINE, CINAHL, PsycInfo, LGBTQ+ Source, and Embase to identify relevant text words and index terms which informed the unique search strategy created for each database (Supplemental Appendix A). On May 25, 2022, the research team conducted a first systematic search. Due to an influx in relevant studies published in the following year, an updated search was performed on July 26, 2023.

### Source of evidence screening and selection

For our first search, we uploaded the five databases’ 1175 identified records to RefWorks for deduplication, then exported the remaining 789 records to Covidence (Veritas Health Innovation, 2022) for review. Per Polanin and colleagues (2019), we developed and pilot-tested an inclusion/exclusion protocol for title and abstract screening. Two reviewers each initially screened 20% of records using this protocol to establish agreement (*k* *=* 0.91). Reviewers then each screened half of the remaining articles. We retrieved manuscripts for all eligible records (*n* = 97) and used a similar protocol for full-text screening (with an added field to indicate reason for exclusion).

**Figure 1.**
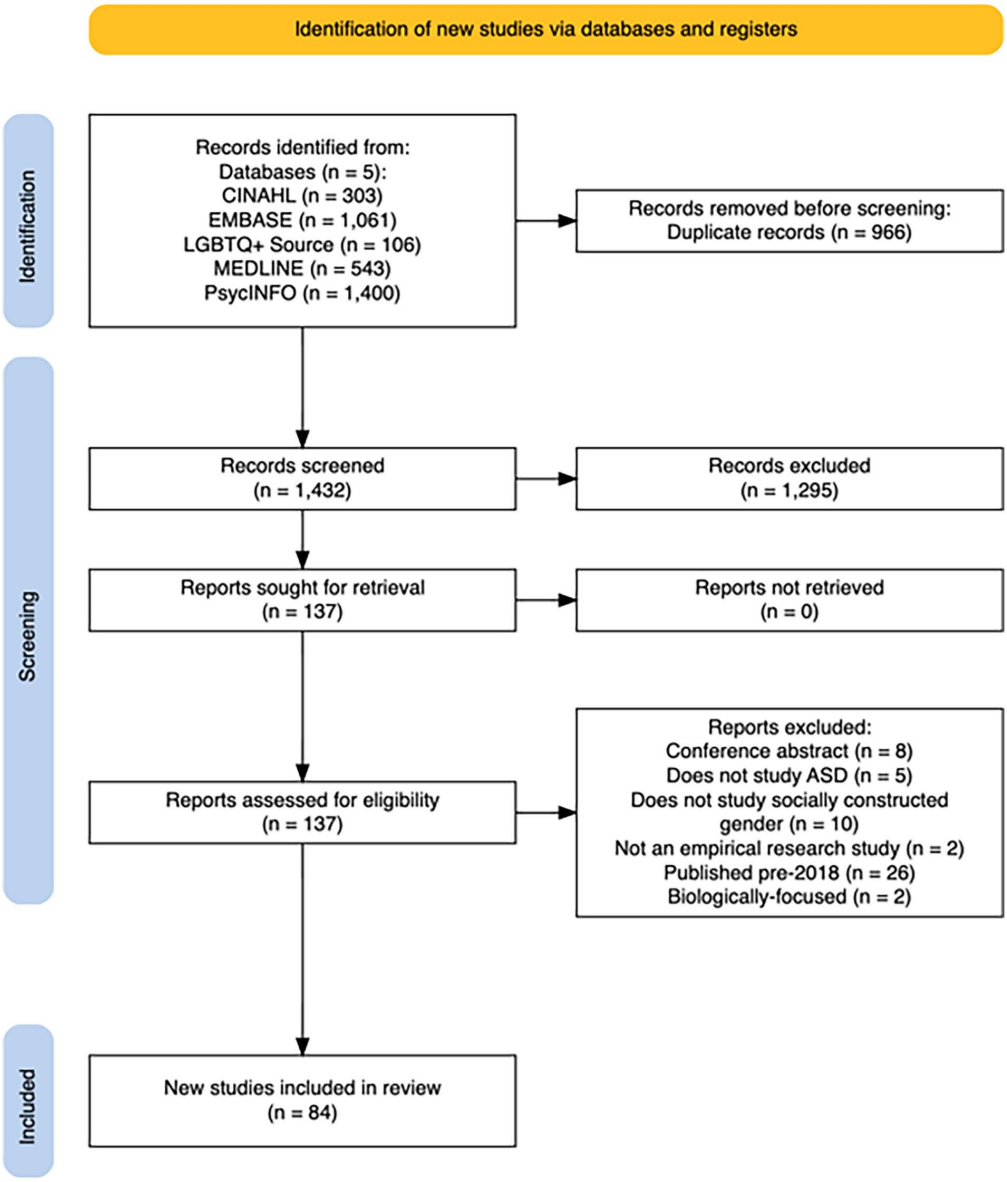
PRISMA flow diagram.

The second search in July 2023 uncovered 643 new, unique records. The first author screened all records at the title and abstract stage. The first and second authors then screened studies for advancement to full-text review (*n* = 133). Twenty-three articles were added to the dataset; Figure 1 (PRISMA flow diagram) maps the process toward our final dataset of 84 studies.

### Data extraction and methodological quality assessment

The first author and three research assistants extracted metadata and key methodological attributes using REDCap (Research Electronic Data Capture; Harris et al., 2009, 2019). After data extraction, reviewers evaluated each study’s robustness and clarity of reporting using quality assessment criteria developed by Kmet and colleagues (2004; see Supplemental Appendix B).

### Data analysis and presentation

Per Kmet and colleagues (2004), quantitative studies were rated on 14 items (maximum score 28) and qualitative studies on 10 items (maximum score 20), with items scored “Yes” (2 points); “Partially” (1 point); “No” (0 points); or “Not applicable” (−2 points from the total maximum score). To enable comparability, we divided each study’s raw score by its maximum score (i.e. the total number of items multiplied by two, minus two points per each “Not applicable” item) to obtain an overall quality percentage score; this ensured that studies with “Not applicable” items were not unjustly penalized. We also divided the sum of each item’s total points by the number of studies applicable to that item, multiplied by two, to obtain mean percentage scores for each item across studies (Tables 3 and 4). For quantitative studies, we considered items scoring >80% across studies to be methodological strengths, items scoring between 60% and 80% as “mixed,” and items scoring <60% as weaknesses. For qualitative studies, we created two categories: strengths (>70%) and weaknesses (⩽70%).

## Results

Systematic searches yielded 63 quantitative (Table 1), 17 qualitative (Table 2), and 4 mixed-method studies. We assessed mixed-method studies as qualitative.

**Table 1.** Characteristics of quantitative studies.

Author (year)	Country	Focus	Research design	Setting	Quality score
Akgul et al. (2018)	Turkey	AT prevalence, executive functioning	Matched case-control	G-DI clinic vs. community	68.18%
Becerra-Culqui et al. (2018)	The United States	Autism prevalence	Matched case-control	Primary care clinic	72.73%
Brandsma et al. (2022)	Netherlands	Peer support intervention	Baseline & follow-up	Autism clinic	66.67%
Bretherton et al. (2021)	Australia	Autism prevalence	Cross-sectional^a^	Community	78.57%
Brunissen et al. (2021)	The United States	G-DI prevalence	Cross-sectional	Community	81.82%
Chang et al. (2022)	Taiwan	“Wish to be of the opposite sex” prevalence	Matched case-control	Psychiatry clinic vs. community	81.82%
Cheung et al. (2018)	Australia	Autism prevalence, service needs/clinical characteristics	Cross-sectional	G-DI clinic & endocrinology clinic	70.00%
Clyde et al. (2022)	The United States	Autism questionnaire validation	Cross-sectional	G-DI clinics	77.27%
Coburn & Williams (2023)	The United States	Differences in communication style	Cross-sectional	Community	95.45%
Cooper et al. (2018)	The United Kingdom	G-DI prevalence	Cross-sectional	Community	77.27%
Corbett et al. (2022)	The United States	“Wish to be of the opposite sex” prevalence, G-DI prevalence	Cross-sectional	Community & autism clinics	86.36%
Fielding & Bass (2018)	The United Kingdom	Autism prevalence, service needs/clinical characteristics	Cross-sectional	G-DI clinic	72.22%
George & Stokes (2018a)	Australia	GD prevalence	Cross-sectional	Community	86.36%
George & Stokes (2018b)	Australia	MH	Cross-sectional	Community	77.27%
Ghassabian et al. (2022)	Netherlands	“Wish to be of the opposite sex” prevalence, GD-I/AT correlation	Baseline & follow-up	Community	86.36%
Greenspan et al. (2023)	The United States	Wellbeing factors	Cross-sectional^a^	Community	90.91%
Hendriks et al. (2022)	The United Kingdom	GD-I/AT correlation	Cross-sectional	Community	81.82%
Heylens et al. (2018)	Belgium	Autism and AT prevalence	Cross-sectional	G-DI clinic vs. community norm	72.73%
Hilton et al. (2022)	Australia	Autism and AT prevalence	Cross-sectional	G-DI clinic vs. community	77.27%
Hisle-Gorman et al. (2019)	The United States	GD prevalence	Matched case-control	Primary care clinic	77.27%
Hull et al. (2020)	The United Kingdom	Levels of autistic camouflaging	Cross-sectional	Community	90.91%
Kahn et al. (2023)	The United States	Co-occurring autism & GD prevalence	Cross-sectional	Pediatric hospitals	90.91%
Kahraman et al. (2021)	Turkey	AT prevalence	Cross-sectional	G-DI clinic vs. community	77.27%
Kallitsounaki & Williams (2020b)	The United Kingdom	GD-I/AT correlation	Cross-sectional	Community	90.91%
Kallitsounaki & Williams (2020a)	The United Kingdom	GD-I/AT correlation	Cross-sectional	Community	86.36%
Kallitsounaki & Williams (2022)	The United Kingdom	GD-I/AT correlation	Matched case-control	Community	81.82%
Kallitsounaki et al. (2021)	The United Kingdom	GD-I/AT correlation	Cross-sectional	Community	80.00%
Kaltiala et al. (2020)	Finland	Effects of HRT on AT	Cross-sectional	G-DI clinic	59.09%
Koffer Miller et al. (2022)	The United States	Service needs/clinical characteristics	Cross-sectional	Community	81.82%
Kung (2020)	The United Kingdom & United States	AT prevalence	Cross-sectional	Local community vs. community norm	86.36%
Kung (2024)	The United Kingdom & United States	MH	Cross-sectional	Community	86.36%
Leef et al. (2019)	Canada	Autism and AT prevalence	Matched case-control	G-DI clinic vs. psychiatry clinic	77.27%
Lehmann et al. (2020)	Ireland	AT prevalence	Cross-sectional^a^	G-DI clinic	77.27%
Mahfouda et al. (2019)	Australia	Autism prevalence	Cross-sectional	G-DI clinic	72.73%
Mazzoli et al. (2022)	Italy	Effects of HRT on AT	Matched longitudinal	G-DI clinics	90.91%
McCallion et al. (2021)	The United Kingdom	Autism prevalence	Cross-sectional	Endocrinology clinic	55.56%
McPhate et al. (2021)	Australia	“Wish to be of the opposite sex” prevalence	Cross-sectional	Psychiatry clinic vs. clinical norm vs. community norm	81.82%
McQuaid et al. (2022)	The United States	Levels of autistic camouflaging	Cross-sectional	Community	90.91%
Morandini et al. (2022)	The United Kingdom	Autism prevalence	Cross-sectional	G-DI clinic	83.33%
Munoz Murakami et al. (2022)	Canada	GD-I/AT correlation	Cross-sectional	Community	86.36%
Murphy et al. (2020)	The United Kingdom	AT prevalence, MH	Cross-sectional	Community	81.82%
Nabbijohn et al. (2019)	Canada	GD-I/AT correlation	Cross-sectional	Community	72.73%
Nobili et al. (2018)	The United Kingdom	AT prevalence	Matched case-control	G-DI clinic vs. community	68.18%
Nobili et al. (2020)	The United Kingdom	Effects of HRT on AT	Baseline & follow-up	G-DI clinic	77.27%
Nunes-Moreno et al. (2022)	The United States	Autism prevalence	Cross-sectional	Primary care clinics	77.27%
Pecora et al. (2020)	Australia	G-DI prevalence	Cross-sectional	Community	90.91%
Pohl et al. (2020)	The United Kingdom	G-DI prevalence	Cross-sectional^a^	Community	77.27%
Reuben et al. (2021)	The United States	MH	Cross-sectional	Community	81.82%
Russell et al. (2021)	The United Kingdom	Effects of HRT on AT	Baseline & follow-up	G-DI clinic	81.82%
Saunders et al. (2023)	The United Kingdom	Autism prevalence	Cross-sectional^a^	Primary care clinics	90.91%
Schiltz et al. (2021)	The United States	GD-I/AT correlation	Cross-sectional	Community	90.91%
Stagg & Vincent (2019)	The United Kingdom	Autism and AT prevalence	Cross-sectional	Community	72.73%
Strang, Jarin, et al. (2018)	The United States	Fertility attitudes	Cross-sectional	GD-I/autism clinic	81.82%
Strang, Anthony, et al. (2021)	The United States	MH	Matched case-control	Community, GD-I clinics & autism clinics	81.82%
Strang, Chen, et al. (2021)	The United States	Executive functioning	Cross-sectional	Community, G-DI clinics & autism clinics	81.82%
Strang, Wallace, et al. (2023)	The United States	G-DI questionnaire validation	Cross-sectional^a^	Community, G-DI clinics & autism clinics	100.00%
Strauss et al. (2021)	Australia	Autism prevalence, MH	Cross-sectional	Community	63.64%
Tollit et al. (2023)	Australia	Service needs/clinical characteristics	Cross-sectional	G-DI clinics	90.91%
van der Miesen, de Vries, et al. (2018)	Netherlands	AT prevalence	Cross-sectional	G-DI clinic vs. clinical norm vs. community norm	72.73%
van der Miesen, Hurley, et al. (2018)	Netherlands	“Wish to be of the opposite sex” prevalence, MH	Cross-sectional	Psychiatry clinic vs. community norm	72.73%
Vermaat et al. (2018)	Netherlands	AT prevalence	Cross-sectional	G-DI clinic vs. community norm	68.18%
Walsh et al. (2018)	Netherlands	AT prevalence	Cross-sectional	Community	86.36%
Warrier et al. (2020)	The United Kingdom	Autism and AT prevalence	Cross-sectional	Community	81.82%

AT = autistic traits; GD = gender dysphoria; G-DI = gender-diverse identities; MH = mental health; HRT = hormone replacement therapy.

a Studies that utilized participatory research methods or community engagement strategies.

**Table 2.** Characteristics of qualitative studies.

Author	Country	Foci	Framework/Lens	Setting	Quality score
Allen-Biddell & Bond (2022)	The United Kingdom	Lived experiences	Thematic analysis/Reflexive	Community	70%
Bruce et al. (2023)	The United Kingdom	Service needs/clinical characteristics	Thematic analysis/Reflexive^a^	Community	100%
Cohen et al. (2023)	The United States	Service needs/clinical characteristics	Not reported	G-DI clinic	70%
Coleman-Smith et al. (2020)	The United Kingdom	Lived experiences	Grounded theory/Social-constructivist	Community; G-DI clinics, autism clinics	100%
Cooper, Butler, et al. (2022)	The United Kingdom	Lived experiences	Multi-perspectival IPA^a^	Community, G-DI clinics, mental health clinics	100%
Cooper, Mandy, Butler, & Russel (2022)	The United Kingdom	Lived experiences	IPA^a^	Community, G-DI clinics, autism clinics	95%
Cooper et al. (2023)	The United Kingdom	Lived experiences	Multi-perspectival IPA^a^	Community, G-DI clinics, autism clinics, mental health clinics	100%
Cooper, Mandy, Russel, & Butler (2022)	The United Kingdom	Lived experiences	IPA^a^	G-DI clinics, autism clinics, mental health clinics	100%
Genovese et al. (2023)	The United States	Lived experiences	Case series	Mental health clinic	35%
Hall et al. (2020)	The United States	Service needs/clinical characteristics	Content analysis	Community	50%
Hillier et al. (2020)	The United States	Lived experiences	Thematic analysis/Realist	Psychotherapy clinic	75%
Kourti & MacLeod (2019)	The United Kingdom	Lived experiences	Applied thematic analysis/Emancipatory^a^	Community	95%
Kuvalanka et al. (2018)	The United States	Lived experiences	Thematic analysis/Essentialist	Community	80%
Maroney & Horne (2022)	The United States	Discrimination experiences	Grounded theory/Critical-constructivist	Community	95%
Mazur (2022)	The United States	Online dating experiences	Content analysis	Community	75%
Pham et al. (2021)	The United States	Service needs/clinical characteristics	Case series	G-DI clinic	35%
Putnam et al. (2023)	The United States	Priorities for autism research	Content analysis	Community	75%
Steinberg et al. (2023)	The United States	Utility of “other” box when querying G-DI	Not reported	Community	70%
Strang, Powers, et al. (2018)	The United States	Lived experiences	Framework analysis/CBPR^a^	G-DI clinic	85%
Strang et al. (2020)	The United States	Clinical intervention	Framework analysis/CBPR^a^	Community, G-DI clinics, autism clinics	85%
Strang, McClellan, et al. (2023)	Multi-national	Development of a GD-I/autism questionnaire	Delphi panel^a^	Community	90%

IPA = interpretative phenomenological analysis; CBPR = community-based participatory research; GD-I = gender-diverse identities.

a Studies that utilized participatory research methods or community engagement strategies.

### Aim 1: foci, research designs, and populations

#### Quantitative studies

Quantitative studies represented 11 countries (majority in the United Kingdom (*n* = 20), the United States (*n* = 19), and Australia (*n* = 10)). Young adults were most studied (*n* = 43), followed by adults (*n* = 33), adolescents (*n* = 32), and children (*n* = 23). Prevalence of autism diagnoses in gender-diverse populations was the most common focus (*n* = 10), followed by correlations between gender diversity and autistic traits in general population samples (*n* = 9), prevalence of autistic traits in gender-diverse populations (*n* = 9), and mental health status among gender-diverse, autistic people (*n* = 7). Other common topics included studying both autism diagnoses and autistic trait prevalence in gender-diverse populations (*n* = 5), the prevalence of gender diversity in autistic populations (*n* = 5), the prevalence of the wish to be the opposite sex in autistic populations (*n* = 5), the effects of hormone therapy on autistic traits (*n* = 4), and service needs/clinical characteristics of gender-diverse, autistic people (*n* = 4). Fifty-one studies were cross-sectional, nine took a matched case-control approach, and four used one baseline and one follow-up assessment. Studies recruited their samples from the general community (*n* = 43), gender identity clinics (*n* = 23), autism clinics (*n* = 6), primary care clinics (*n* = 4), psychiatry clinics (*n* = 4), endocrine clinics (*n* = 2), and pediatric hospitals (*n* = 1). Community convenience sampling was the most common sampling method (*n* = 26), followed by consecutive referral (*n* = 19), representative (*n* = 9), clinical convenience (*n* = 3), and purposive (*n* = 2) sampling. Six studies used participatory research methods or patient/community engagement strategies.

#### Qualitative studies

Qualitative studies were conducted in the United States (*n* = 12), the United Kingdom (*n* = 8), and internationally (*n* = 1), with sample sizes ranging from 3 to 1527 (median = 21). Twelve studies included adults, fifteen included young adults, and six included adolescents. In addition, five studies included clinicians, four included parents, and one included researchers. Eleven studies explored the lived experiences of autistic, gender-diverse people and four explored the clinical characteristics and service needs of autistic, gender-diverse people. The remaining six explored minority stress, experiences with online dating, opinions on autism research priorities, best practices in querying gender on surveys, developing a gender diversity and autism questionnaire, and piloting a clinical intervention. The most common methodological frameworks used included thematic analysis (*n* = 5), interpretative phenomenological analysis (*n* = 4), qualitative content analysis (*n* = 3), framework analysis (*n* = 2), and grounded theory (*n* = 2). Studies used interviews (*n* = 13), qualitative surveys (*n* = 4), interviews and quantitative surveys (*n* = 3), focus groups (*n* = 2), and medical chart history (*n* = 2). Nine studies used participatory research methods or patient/community engagement strategies.

Ten studies recruited only community samples, three only gender clinic samples, one only mental health clinics, and one only psychotherapy clinics. The remaining six recruited from a mixture of community and gender, autism, and mental health clinics. All studies used a form of convenience sampling (e.g. purposive, snowball).

### Aim 2: key terms and definitions

This section discusses quantitative and qualitative studies together (for detailed tables of results, see Supplemental Appendix C). For gender identity terminology, 63% of studies reported fully the gender with which participants currently identified. Of those reporting gender, the most common term was “gender identity” (*n* = 43); for gender identity options, most studies (*n* = 48) included nonbinary categories. Qualitative studies often provided open-ended text entries, while quantitative studies usually provided predetermined options. Regarding the reporting of sexes, 83% of studies reported participants’ SAB; however, most studies (*n* = 61) provided only “male” and “female” options, with no consideration for intersex status. Most used a sex-based label such as SAB (*n* = 53) while the remaining used a gender-based term (e.g. “assigned gender”). Slightly more studies used person-first (54%) than identity-first language to describe autism. A wide range of terms described different gender-diverse sub-populations; the most common being “people with gender dysphoria” (29%).

There were diverse approaches to measuring concepts related to gender identity (Supplemental Appendix D). Of the 21 studies measuring aspects of gender diversity (e.g. variance, dysphoria, expression) in autistic individuals using standardized tools, seven interpreted scores continuously (i.e. participants’ total scores), eight categorically (e.g. number of participants scoring above cutoff), and six adopted both continuous and categorical approaches. Gender-related variables were commonly assessed using study-specific self-report surveys (*n* = 22), inferred based on gender identity clinic referrals (*n* = 12), or evaluated using clinician interviews that derived GD diagnoses using the *Diagnostic and Statistical Manual of Mental Disorders, Fifth Edition*, (*DSM*-5) criteria (*n* = 9).

Similarly diverse methods were used to measure autism-related concepts (Supplemental Appendix D). Thirty studies measured autism categorically, 13 continuously, and 20 used both categorical and continuous approaches. Autism-related variables were most commonly measured using study-specific self-report surveys (*n* = 18), review of electronic medical records (*n* = 14), and self-report or informant report questionnaires (e.g. 22 studies used a version of the Autism Spectrum Quotient). Only seven studies used clinician or diagnostician-administered measures, and only three used gold-standard tools (i.e. Autistm Diagnostic Interview-Revised (ADI-R), Autism Diagnostic Observation Schedule-2 (ADOS-2)).

### Aim 3: methodological strengths and weaknesses

Tables 1 (quantitative) and 2 (qualitative and mixed-method) provide percentage scores for each study per the methodological assessment designed by Kmet and colleagues (2004). Tables 3 (quantitative) and 4 (qualitative and mixed-method) present the endorsement frequencies for scores of “Yes (2),” “Partially (1),” and “N/A (0)” for each methodological item.

**Table 3.** Quantitative quality assessment (Kmet et al., 2004).

Criterion	Yes	Partially	NA	Item score
1. Objective	58	5	0	96.03%
2. Design	42	21	0	83.33%
3. Sampling	23	39	0	67.46%
4. Participant characteristics	20	34	0	58.73%
6. Blinding	0	0	62	0%
8. Measures	28	23	0	62.70%
9. Power	17	31	2	53.28%
10. Analysis	60	2	1	98.39%
11. Estimates of variance	58	0	3	96.67%
12. Controlled for confounding	46	8	6	87.72%
13. Results	55	8	0	93.65%
14. Conclusions	47	16	0	87.30%

If a certain criterion was not at all applicable to a given study (e.g. blinding in a correlational study), then this was given a score of “NA.” If a criterion was applicable but not met, and there was a justifiable reason for why this was so, no penalty was given (e.g. if a study stated that it could not report gender identity because the sample was too young, then the full 2 points for “Participant Characteristics” were still awarded). Items 6 and 7 were excluded from this table as these items were scored as NA for all studies.

**Table 4.** Qualitative quality assessment (Kmet et al., 2004).

Criterion	Yes	Partially	Item score
1. Objective	20	1	97.62%
2. Design	20	1	97.62%
3. Context	19	1	92.86%
4. Connection to theories	13	6	76.19%
5. Sampling	12	8	76.19%
6. Data collection	17	3	88.1%
7. Analysis	16	3	83.33%
8. Verification	11	0	52.38%
9. Conclusions	19	2	95.24%
10. Reflexivity	8	1	40.48%

#### Methodological quality of quantitative studies

The overall quality of the 63 quantitative studies ranged from 55.56% to 100%, with an average quality score of 80.19%. Using the cut point of 75% suggested by Kmet and colleagues (2004) for an adequate study, we can infer quality was satisfactory on average. Of the nine topics considered by four or more studies, studies examining the correlation between gender diversity and autistic traits had the highest average quality (84.14%), followed by studies examining the prevalence of gender-diverse identities and gender dysphoria in autistic people (82.73%), and those examining the wish to be the opposite gender in autistic people (81.82%). Studies examining clinical characteristics and service needs of autistic, gender-diverse people had a moderate average score of 78.73%. Among studies examining autism in gender-diverse people, those reporting on autistic traits and on autism diagnoses also fell in the moderate range (76.26% and 73.70%, respectively); but notably, none employed a gold-standard autism diagnostic tool. Studies examining effects of hormone replacement therapy on autistic traits had the lowest average quality (66.67%). Average study quality varied somewhat by autism measurement, with studies using clinician-administered tools scoring highest (83.12%), followed by adult self-report surveys (e.g. of diagnostic history; 83.04%) or measures (e.g. Autism Spectrum Quotient; 82.33%), youth self-report surveys (78.79%) and measures (78.28%), and parent-report surveys (77.92%) and measures (77.27%). See Supplemental Appendix D for a complete list of measures used.

Overall strengths (Table 3) included clearly defined research questions, comprehensive data analytic strategies, appropriate estimates of variance, controlling for confounds, and study design. A design weakness was a tendency to make comparisons between gender-diverse and cisgender individuals without a comparative study design (e.g. informally comparing observed prevalence rates to previously published data); however, there were several robust matched case-control studies. Nine studies used comparison groups without accounting for the confounding role of service seeking (i.e. comparing autistic characteristics in a treatment-seeking GD sample to the general population). Most, but not all studies controlled for age and SAB. The majority reported results comprehensively and made logical conclusions that discussed theoretical and clinical implications.

We found mixed results for sampling strategies, methods used to confirm participant eligibility (i.e. participant characteristics), and measures. Most frequent reasons for partial or no credit on these items included using non-representative sampling, lacking clarity regarding inclusion/exclusion criteria, not reporting a sampling frame, or confirming autism only by asking the participant whether they had ever been formally diagnosed. Furthermore, measures tended to lack robustness such that they were not validated in the sample population or altered by the research team in ways that compromised validity (e.g. administering a single item from a multi-item inventory). No studies measuring prevalence of autism among gender-diverse samples used a validated diagnostic tool.

Other areas of weakness included participant characterization and discussion of statistical power, which was lacking in several studies despite small sample sizes and/or sample stratification. Complete reporting of participant characteristics was absent from several studies: notably, four did not report SAB, and 24 did not report gender identity but provided a quantitative measurement of gender variance.

#### Methodological quality of qualitative studies

Overall, scores of qualitative studies ranged from 35% to 100%, with an average quality score of 80% (Table 2). This suggests little differences in quality between the qualitative and quantitative studies. In the following summary, percentage scores refer to *item* scores (i.e. derived from the sum of all studies’ quality scores for a given item), not to the percentage of studies who achieved full points on the item.

Regarding strengths, all but one study had a fully defined research question/objective and an adequately described research design (Table 4). Most fully described the research background, context, premises, and data collection/analysis methods and made data-driven conclusions. Weaknesses arose in study sampling, connecting findings to a broader framework or knowledge base, verification procedures, and reflexivity. Several samples had limited generalizability and were poorly characterized. Although maintaining methodological integrity by triangulating data interpretations is emphasized in qualitative research (Levitt et al., 2018), only half of the studies verified their data through participant feedback, and most did not use reflexivity procedures (i.e. a self-reflection journal, interrogating personal assumptions).

## Discussion

The intersection of autism and gender diversity is a burgeoning area of study, with 84 studies published between January 2018 and July 2023. Our methodological assessments demonstrate that, despite overall acceptable quality, these studies have specific, recurring threats to generalizability and validity that must be addressed in future work, which is concerning but unsurprising given the limited infrastructure (e.g. validated measures, construct consensus, recruitment networks) available to support research in historically marginalized populations (Popejoy & Fullerton, 2016).

A strength of the recent literature is its expanding scope. For example, while 86% of 2018 studies focused on prevalence, this was the case for only 36% of 2022 studies, which more broadly covered topics such as the effects of hormone therapy on autistic characteristics, the wellbeing of gender-diverse, autistic people, and access to health services. This expansion to health needs and services research is important because many gender-diverse, autistic participants report decreased access to, and satisfaction with, healthcare services (e.g. Hall et al., 2020; Strang, Powers, et al., 2018). Another strength lies in studies that highlight the voices and experiences of gender-diverse, autistic people by employing participant-oriented methodologies (e.g. framework analysis) or patient engagement strategies (e.g. community advisory panels, patient co-authors). These methods were more prevalent among qualitative studies (*n* = 9) than among quantitative ones (*n* = 6).

Nonetheless, many weaknesses of earlier studies on autism and gender diversity remain, including lack of comparison groups, inconsistent terminology, low measurement specificity and validity, and an absence of power analysis (see Manjra & Masic, 2022). Furthermore, our review highlights novel challenges, such as poorly controlled comparisons, unreliable confirmation of eligibility criteria, limited reporting of SAB and gender identity, and a lack of reflexivity and verification procedures. For example, several studies compared autistic or gender-dysphoric participants recruited from medical clinics with general population samples, making it impossible to assess whether differences were due to the clinical nature of the autistic/gender-dysphoric group or to the presence of autism/gender dysphoria. The lack of verification procedures and self-reflexivity in qualitative studies is equally concerning. Dark moments in psychology’s history—such as sexually-diverse people being psychopathologized—have partially stemmed from limited critical reflection about power relations between the researcher and the researched (Danaher et al., 2013). Self-reflexive activities can unmask tacit assumptions and avoid replicating harmful power relations (Mortari, 2015), especially when working with marginalized populations (Danaher et al., 2013).

Studies also demonstrated a lack of precision and clarity in key concepts and operational definitions. For example, using “transgender” as an umbrella term when reporting demographics erases the nuances that differentiate binary and nonbinary identities. In addition, a “transgender sample” at times referred to people formally diagnosed with GD, while at other times, it referred to those who self-identified as gender-diverse. Health indicators and demographics differ significantly between binary transgender (i.e. trans male and trans female) and nonbinary people (Scandurra et al., 2019), and there is a high correlation between GD severity and mood/anxiety disorders (Sood et al., 2021). Thus, it is important that studies clearly delineate GD versus self-identified gender-diverse samples and binary versus nonbinary samples. Studies also often used “gender identity” as a dichotomous categorical variable: transgender and cisgender. This practice is problematic because “transgender” and “cisgender” do not constitute gender identities; rather, they describe whether a participant’s gender is congruent with their SAB. Given the likely existence of nonbinary and agender identities among these groups simply labeled “transgender,” using this binary language decreases precision in results and risks disregarding heterogeneity in non-cisgender people’s experiences and outcomes (Scandurra et al., 2019). Finally, studies often used “typically developing,” “control,” “neurotypical,” and “non-autistic” interchangeably; this practice is discouraged because “typically developing” assumes participants do not have other neurodevelopmental diagnoses, while “non-autistic” (or the preferred term “allistic”) does not (Bottema-Beutel et al., 2020). As research suggests autism is not the only neurodevelopmental disorder associated with gender diversity (e.g. McPhate et al., 2021; Nabbijohn et al., 2019), whether participants with non-autism neurodevelopmental diagnoses are included may impact results. Agreement in the operationalization of these terms will be essential to generalize and integrate findings across studies.

Regarding sample characteristics, some studies reported participants’ SAB but not gender identity. These studies often included a quantitative measure of gender diversity, such as the Gender Identity/Gender Dysphoria Questionnaire for Adolescents and Adults (GIDYQ-AA) or Gender Identity Questionnaire for Children (GIQC). These screeners, however, do not adequately replace self-reported gender identity. Rather than directly capturing any particular gender identity, they assess common characteristics of gender nonconformity and gender dysphoria to estimate the degree to which a participant diverges from traditional gender norms. The use of pre-existing medical records or a very young sample may have precluded the collection of valid gender identity information in some studies; however, the omission of self-reported gender identity in research using adult self-report questionnaires is an avoidable issue that runs the risk of discounting participants’ self-knowledge and complicates study replication.

Studies with GD samples were often unclear about whether participants met *DSM*/ICD criteria; sometimes, researchers simply classified participants as GD if they identified as gender diverse or if they had been referred to a gender identity clinic. This is a problematic overgeneralization, given that not all gender-diverse people experience clinically-significant gender-related distress. In fact, for many, the social stigma associated with having a gender-diverse identity causes significantly more psychological distress than GD itself (Bockting, 2009). The validity of using a single item (e.g. “Does your child ever wish to be of the opposite sex?”; “Would you rather be treated as the opposite sex?”) to assess gender diversity is questionable and yet was the standalone measure of gender diversity used in five studies. Some researchers have operationalized these singular items as tapping gender variance but then interpret findings of endorsement of these items among autistic people as indicative of gender dysphoria. This is of concern as researchers posit that this item is not exclusively endorsed by people with gender dysphoria, and autistic people may endorse it for reasons other than underlying GD (Turban & van Schalkwyk, 2018). For example, Turban and Van Schalkwyk (2018) state that parents may interpret an assigned-male-at-birth (AMAB) autistic child’s focused interests in “feminine” pursuits as representative of their gender identity, leading the parents to endorse the “wish to be of opposite sex” despite the child having no interest in adopting a feminine gender identity.

Characterization of autism was also often problematic, with many studies relying on self-report of a community diagnosis, medical chart review, or non-diagnostic screening tools, which often demonstrate low specificity, particularly in those with co-occurring mental health conditions (Fombonne, 2018). Relying on screeners in gender-diverse people may be especially problematic: In their review, Thrower and colleagues (2020) noted that scores on autism screeners may be falsely inflated by social injustices experienced by gender-diverse people (e.g. social rejection, isolation), which may result in characteristics similar to those seen in autistic samples (e.g. difficulties with social interaction, inability to relate with peers; Turban & van Schalkwyk, 2018). Concerningly, the use of autism diagnostic instruments was rare among studies in our sample; exceptions included Chang et al. (2022), Corbett et al. (2022), and four studies by Strang, Powers et al. (2018, Strang and colleagues, 2020; Strang, Anthony, et al., 2021; Strang, Chen, et al., 2021). Overall, we found studies using clinician-administered and adult-report surveys/measures had slightly higher average quality scores than those using youth- or parent-report surveys/measures alone.

Overall, studies measuring gender diversity among autistic people were of moderate to high quality. This particular research sub-topic has many promising developments that will likely lead to stronger measurement validity and reliability—for example, the Gender Self-Report by Strang, Wallace, et al. (2023) is a newly validated measure of gender diversity that was designed specifically with autistic individuals’ needs in mind. On the other hand, lower quality was observed in studies of autistic traits and autism prevalence among gender-diverse people—particularly concerning was the complete absence of validated autism diagnostic tools from these studies. This area of research could be improved by using tools like the ADOS-2 or ADI-R, conducting validation studies of existing autism trait questionnaires using gender-diverse samples, and creating new autism trait questionnaires in collaboration with autistic, gender-diverse patient partners.

### Limitations

Quality assessment was limited to a predefined set of criteria (some of which were not applicable to studies’ designs in this review). Although we took steps to ensure the comparability of studies, nonetheless there may be methodological strengths and weaknesses not captured by the protocol.

## Conclusion

In conclusion, recent studies on gender diversity/GD and autistic characteristics/autism, although growing in size and kind, remain hindered by many of the same challenges identified in earlier reviews. Prevailing limitations (e.g. the use of measures not validated for the sample population) may reflect a more systematic and pervasive set of issues faced by those studying these constructs. The under-resourced nature of research on marginalized people has led to a paucity of appropriately validated measures, difficulty obtaining representative and generalizable samples, and small sample sizes. As such, low quality scores may reflect the inadequacy of current infrastructure to support robust research. Recommendations are offered below to address the ongoing issues identified and ensure future work can better represent the needs and inform healthcare policy and practices for gender-diverse, autistic people.

1. Continue to **expand the research scope** by moving away from prevalence studies to broader questions about health and wellbeing: do autistic, gender-diverse people experience high quality of life? If not, what systemic factors are preventing this? How do issues that affect both the autistic and gender-diverse communities separately (e.g. bullying, healthcare barriers, stigma) compound for those holding both identities (Crenshaw, 1989)?
2. **Control for clinical status** by ensuring that when autistic and gender-diverse participants are recruited from clinical settings, comparison groups are as well.
3. Use participant-centered **data verification** methods and engage in **self-reflexive processes** by examining how biases affect data collection and interpretation (Danaher et al., 2013).
4. **Community collaboration** among the qualitative studies was a notable strength; quantitative studies would do well to follow suit (den Houting et al., 2021). Autistic and gender-diverse patient partners are imperative to developing study methods: They can provide input regarding how language, terminology, and data collection modalities can be more accessible.
5. **Establish consensus regarding the meaning and operationalization of terms** (e.g. gender identity, variance, diversity, dysphoria, congruence) and ensure more **precise sample characterization**. When sample size allows, strive for precision by going beyond a trans-cis dichotomy to differentiate between trans men/women, cis men/women, and nonbinary people.
6. **Recruit from diverse settings.** Strang, Anthony, et al. (2021; Strang, Chen, et al., 2021) intentionally recruited from gender identity clinics and autism clinics, through social media, and the community, resulting in samples that were not as biased toward treatment-seeking individuals.
7. **Conduct validation studies** of autism screeners among gender-diverse samples, and of gender identity questionnaires among autistic samples. Ideally, **create novel tools** in collaboration with the autistic and gender-diverse community. For example, Strang McClellan, et al. (2023; Strang, Wallace, et al., 2023) developed the Gender Self-Report and the Gender Diversity and Autism Questionnaire using methods that engaged and consulted with gender-diverse and autistic people throughout their design to ensure their accessibility and relevance.

## Supplemental Material

sj-docx-1-aut-10.1177_13623613241245595 – Supplemental material for Research methods at the intersection of gender diversity and autism: A scoping review Supplemental material, sj-docx-1-aut-10.1177_13623613241245595 for Research methods at the intersection of gender diversity and autism: A scoping review by Em JE Mittertreiner, Elise Ng-Cordell, Alana J McVey and Connor M Kerns in Autism

sj-docx-2-aut-10.1177_13623613241245595 – Supplemental material for Research methods at the intersection of gender diversity and autism: A scoping review Supplemental material, sj-docx-2-aut-10.1177_13623613241245595 for Research methods at the intersection of gender diversity and autism: A scoping review by Em JE Mittertreiner, Elise Ng-Cordell, Alana J McVey and Connor M Kerns in Autism
