# Supplementary material for: Research methods at the intersection of gender diversity and autism: A scoping review
Source: Autism. 2024 Apr 25;28(12):2953–69. doi: 10.1177/13623613241245595 (PMC11575102; doi:10.1177/13623613241245595)
Supplement: sj-docx-2-aut-10.1177_13623613241245595 – Supplemental material for Research methods at the intersection of gender diversity and autism: A scoping review [file sj-docx-2-aut-10.1177_13623613241245595.docx]

**Research methods at the intersection of gender diversity and autism: A scoping review**

**Supplementary Material**

Appendix A. Scoping Review Search Strategies

Appendix B. Quality Assessment Items

Appendix C. Gender, Sex, and Autism Terminology in Quantitative and Qualitative Studies

Appendix D. Gender Diversity and Autism Measures in Quantitative Studies

**Appendix A**

Search Strategies

**OVID MEDLINE**

1 exp Gender Identity/ or exp Sex Reassignment Procedures/ or exp "Sexual and Gender Minorities"/ or "Health Services for Transgender Persons"/ or Transsexualism/ or Gender Dysphoria/ or "Sexual and Gender Disorders"/ or Transvestism/

2 (transgender* or transmasculine or transfeminine or transmale? or transfemale? or transm#n or transwom#n or transsex* or trans-sex* or intersex* or inter-sex*or crossgender* or cross-gender* or F2M or M2F or sex change or sex* reassignment or sex* reversal or sex* transition or sex* transform* or gender disorder or nonbinary or non-binary or genderdivers* or genderqueer or agender or genderfluid or two-spirit* or 2-spirit* or (gender adj (identi* or self-concept? or reassignment or therap* or clinic? or affirm* or service? or minorit* or dysphori? or nonconform* or varian* or queer or spectrum? or divers* or role? or incongruen* or congruen* or atypical* or norm* or non-norm* or expression* or creativ* or fluid* or transition* or transform*)) or (trans* adj (boy? or girl? or m#n or wom#n or male? or female? or masculine or feminine or people or person*)) or ((hormon* or testosterone or estrogen) adj (therap* or treatment* or medication*))).ti,ab,kf.

3 exp Child Development Disorders, Pervasive/

4 (autis* or asperger* or kanner* or pervasive development* disorder* or pdd or pdd-nos or neurodivers* or asd).ti,ab,kf.

5 (1 or 2) and (3 or 4)

6 5 not (review or editorial or letter or systematic review or comment or case reports or meta analysis).pt.

**OVID EMBASE**

1 exp Gender Diversity/ or exp "Transgender and Gender Nonbinary"/ or exp "Sexual and Gender Minority"/ or femininity/ or masculinity/ or exp Gender Identity/ or exp Gender Dysphoria/ or Transsexuality/ or Sex Reassignment/ or Sex Role/ or Cross-Dressing/ or Transphobia/

2 (transgender* or transmasculine or transfeminine or transmale? or transfemale? or transm#n or transwom#n or transsex* or trans-sex* or intersex* or inter-sex*or crossgender* or cross-gender* or F2M or M2F or sex change or sex* reassignment or sex* reversal or sex* transition or sex* transform* or gender disorder or nonbinary or non-binary or genderdivers* or genderqueer or agender or genderfluid or two-spirit* or 2-spirit* or (gender adj (identi* or self-concept? or reassignment or therap* or clinic? or affirm* or service? or minorit* or dysphori? or nonconform* or varian* or queer or spectrum? or divers* or role? or incongruen* or congruen* or atypical* or norm* or non-norm* or expression* or creativ* or fluid* or transition* or transform*)) or (trans* adj (boy? or girl? or m#n or wom#n or male? or female? or masculine or feminine or people or person*)) or ((hormon* or testosterone or estrogen) adj (therap* or treatment* or medication*))).ti,ab,kf.

3 exp autism/ or exp autism assessment/

4 (autis* or asperger* or kanner* or pervasive development disorder* or pdd or pdd-nos or neurodiver* or asd).ti,ab,kf.

5 (1 or 2) and (3 or 4)

6 5 not (conference abstract status.mp. or review.pt. or editorial.pt. or letter.pt. or note.pt. or case report/ or conference paper.pt. or short survey.pt.)

**EBSCO PsycINFO**

S6 ((S1 OR S2) AND (S3 OR S4)) NOT S5

S5 PT ("Book" or "Edited Book" or "Authored Book" or "Dissertation Abstract" or "Case Studies" or "Practice Guideline") OR PZ ("Letter" or "Comment/Reply" or "Review-Book" or "Chapter" or "Review-Media" or "review-Other" or "Editorial" or "Clarification" or "Dissertation" or "Column/Opinion") OR MR "Meta analysis" or "Metasynthesis" or "Systematic review" or "Literature review" or "Brain Imaging"

S4 TI (autis* or asperger* or kanner* or "pervasive development* disorder*" or pdd or pdd-nos or neurodiver* or asd) OR AB (autis* or asperger* or kanner* or "pervasive development* disorder*" or pdd or pdd-nos or neurodiver* or asd)

S3 DE (“Autism Spectrum Disorders" OR "Autistic Traits" OR "Applied Behavior Analysis" OR "Neurodiversity”)

S2 TI (transgender* OR transmasculine OR transfeminine OR transmale? OR transfemale? OR transm?n OR transwom?n OR transsex* OR trans-sex* OR intersex* OR inter-sex*or crossgender* OR cross-gender* OR F2M OR M2F OR sex change OR sex* reassignment OR sex* reversal OR sex* transition OR sex* transform* OR gender disorder OR nonbinary OR non-binary OR genderdivers* OR genderqueer OR agender OR genderfluid OR two-spirit* OR 2-spirit* OR (gender W1 (identi* OR self-concept? OR reassignment OR therap* OR clinic? OR affirm* OR service? OR minorit* OR dysphori? OR nonconform* OR varian* OR queer OR spectrum? OR divers* OR role? OR incongruen* OR congruen* OR atypical* OR norm* OR non-norm* OR expression* OR creativ* OR fluid* OR transition* OR transform*)) OR (trans* W1 (boy? OR girl? OR m?n OR wom?n OR male? OR female? OR masculine OR feminine OR people OR person*)) OR (hormon* OR testosterone OR estrogen W1 (therap* OR treatment* OR medication*))) OR AB (transgender* OR transmasculine OR transfeminine OR transmale? OR transfemale? OR transm?n OR transwom?n OR transsex* OR trans-sex* OR intersex* OR inter-sex*or crossgender* OR cross-gender* OR F2M OR M2F OR sex change OR sex* reassignment OR sex* reversal OR sex* transition OR sex* transform* OR gender disorder OR nonbinary OR non-binary OR genderdivers* OR genderqueer OR agender OR genderfluid OR two-spirit* OR 2-spirit* OR (gender W1 (identi* OR self-concept? OR reassignment OR therap* OR clinic? OR affirm* OR service? OR minorit* OR dysphori? OR nonconform* OR varian* OR queer OR spectrum? OR divers* OR role? OR incongruen* OR congruen* OR atypical* OR norm* OR non-norm* OR expression* OR creativ* OR fluid* OR transition* OR transform*)) OR (trans* W1 (boy? OR girl? OR m?n OR wom?n OR male? OR female? OR masculine OR feminine OR people OR person*)) OR (hormon* OR testosterone OR estrogen W1 (therap* OR treatment* OR medication*)))

S1 DE "Gender Dysphoria" OR DE "Gender Nonconforming" OR DE "Gender Transition" OR DE "Gender Nonbinary" OR DE "Gender Expression" OR DE "Gender Reassignment" OR DE "Gender Identity" OR DE "Gender Nonbinary" OR DE "Gender Nonconforming" OR DE "Gender Affirming Care" OR DE "Hormone Therapy" OR DE "Personal Pronouns" OR DE "Transsexualism" OR DE "Transgender" OR DE "Transvestism" OR DE "Intersex" OR DE "Affirmative Therapy"

**EBSCO CINAHL**

S8 ((S1 OR S2 OR S3 OR S4) AND (S5 OR S6)) NOT S7

S7 PT "Systematic Review" or "Response" or "Commentary" or "Review" or "Practice guidelines" or "Corrected Article" or "Book review" or "Meta synthesis"

S6 TI (autis* or asperger* or kanner* or pervasive development disorder* or pdd or pdd-nos or neurodiver* or asd) OR AB (autis* or asperger* or kanner* or pervasive development disorder* or pdd or pdd-nos or neurodiver* or asd)

S5 MH “Child Development Disorders, Pervasive+”

S4 TI (transgender* OR transmasculine OR transfeminine OR transmale* OR transfemale* OR transm?n OR transwom?n OR transsex* OR transvest* OR nonbinary OR non-binary OR genderdiver* OR genderqueer*) OR AB (transgender* OR transmasculine OR transfeminine OR transmale* OR transfemale* OR transm?n OR transwom?n OR transsex* OR transvest* OR nonbinary OR non-binary OR genderdiver* OR genderqueer*)

S3 TI (trans W1 (boy OR girl OR man OR woman OR male OR female OR masculine OR feminine)) OR AB (trans W1 (boy OR girl OR man OR woman OR male OR female OR masculine OR feminine))

S2 TI (gender W1 (identi* OR self-concept OR reassignment OR therapy OR clinic OR service OR minority OR dysphori#? OR nonconform* OR varian* OR queer OR spectrum OR diver* OR role OR incongruen* OR congruen* OR atypical* OR norm* OR non-norm* OR expression OR creativ* OR fluid OR transition)) OR AB (gender W1 (identi* OR self-concept OR reassignment OR therapy OR clinic OR service OR minority OR dysphori#? OR nonconform* OR varian* OR queer OR spectrum OR diver* OR role OR incongruen* OR congruen* OR atypical* OR norm* OR non-norm* OR expression OR creativ* OR fluid OR transition))

S1 (MH "Gender-Nonconforming Persons+") OR (MH "Gender Affirmation Procedures+") OR (MH "Gender Affirming Care") OR (MH "Gender Affirmation Surgery") OR (MH "Sexual and Gender Minorities+") OR (MH "Gender Transition") OR (MH "Gender Identity+") OR (MH "Gender Dysphoria") OR (MH "Nonbinary Persons") OR (MH "Transgender Persons+") OR (MH "Transphobia") OR (MH "Trans Women") OR (MH "Trans Men")

**EBSCO LGBTQ+ Source**

S6 (S1 OR S2 OR S3 OR S4) AND S5

S5 TX TI (“Autism Spectrum Disorders" OR "Autistic Traits" OR "Neurodiversity” OR “autis*” or “asperger*” or "pervasive development* disorder*" or “pdd” or “pdd-nos” or neurodiver* or “asd”) OR AB (“Autism Spectrum Disorders" OR "Autistic Traits" OR "Neurodiversity” OR “autis*” or “asperger*” or "pervasive development* disorder*" or “pdd” or “pdd-nos” or neurodiver* or “asd”)

S4 TI (transgender* OR transmasculine OR transfeminine OR transmale* OR transfemale* OR transm?n OR transwom?n OR transsex* OR transvest* OR nonbinary OR non-binary OR genderdiver* OR genderqueer*) OR AB (transgender* OR transmasculine OR transfeminine OR transmale* OR transfemale* OR transm?n OR transwom?n OR transsex* OR transvest* OR nonbinary OR non-binary OR genderdiver* OR genderqueer*)

S3 TI (trans W1 (boy OR girl OR man OR woman OR male OR female OR masculine OR feminine)) OR AB (trans W1 (boy OR girl OR man OR woman OR male OR female OR masculine OR feminine))

S2 TI (gender W1 (identi* OR self-concept OR reassignment OR therapy OR clinic OR service OR minority OR dysphori#? OR nonconform* OR varian* OR queer OR spectrum OR diver* OR role OR incongruen* OR congruen* OR atypical* OR norm* OR non-norm* OR expression OR creativ* OR fluid OR transition)) OR AB (gender W1 (identi* OR self-concept OR reassignment OR therapy OR clinic OR service OR minority OR dysphori#? OR nonconform* OR varian* OR queer OR spectrum OR diver* OR role OR incongruen* OR congruen* OR atypical* OR norm* OR non-norm* OR expression OR creativ* OR fluid OR transition))

S1 DE "GENDER affirmation surgery" OR DE "FEMALE-to-male surgery" OR DE "MALE-to-female surgery" OR DE "GENDER benders (Gender expression)" OR DE "GENDER bending (Gender expression)" OR DE "GENDER dysphoria" OR DE "GENDER euphoria" OR DE "GENDER expression" OR DE "ANDROGYNY (Psychology)" OR DE "CROSS-dressing" OR DE "GENDER nonconformity" OR DE "GENDER identity" OR DE "ANDROGYNOUS identity" OR DE "FEMININE identity" OR DE "GENDER transition" OR DE "INTERSEXUAL identity" OR DE "MASCULINE identity" OR DE "THIRD gender" OR DE "TRANSGENDER identity" OR DE "TRANSSEXUALISM" OR DE "GENDER spectrum" OR DE "GENDER-neutral pronouns" OR DE "GENDER-nonconforming people" OE DE "HEALTH of transgender people" or DE "TRANSGENDER people" OR DE "TRANS men" OR DE "TRANS women" OR DE “HORMONE therapy”

**Appendix B**

Scoring Protocol for Kmet et al.’s (2004) Quality Assessment

| **Item** | **Quality scoring of quantitative studies (Kmet et al., 2004)** | **Quality scoring of qualitative and mixed-methods studies**  **(Kmet et al., 2004)** |
| --- | --- | --- |
| 1 | Question or objective sufficiently described? | Question / objective clearly described? |
| 2 | Design evident and appropriate to answer study question? | Design evident and appropriate to answer study question? |
| 3 | Method of subject selection (and comparison group selection, if applicable) or source of information/input variables (eg., for decision analysis) is described and appropriate. | Context for the study is clear? |
| 4 | Subject (and comparison group, if applicable) characteristics or input variables/information (e.g., for decision analyses) sufficiently described? | Connection to a theoretical framework / wider body of knowledge? |
| 5 | If random allocation to treatment group was possible, is it described? | Sampling strategy described, relevant and justified? |
| 6 | If interventional and blinding of investigators to intervention was possible, is it reported? | Data collection methods clearly described and systematic? |
| 7 | If interventional and blinding of subjects to intervention was possible, is it reported? | Data analysis clearly described, complete and systematic? |
| 8 | Outcome and (if applicable) exposure measure(s) well defined and robust to measurement / misclassification bias? Means of assessment reported? | Use of verification procedure(s) to establish credibility of the study? |
| 9 | Sample size appropriate? | Conclusions supported by the results? |
| 10 | Analysis described and appropriate? | Reflexivity of the account? |
| 11 | Some estimates of variance (e.g., confidence intervals, standard errors) is reported for the main results/outcomes (i.e., those directly addressing the study question/objective upon which the conclusions are based)? | NA |
| 12 | Controlled for confounding? | NA |
| 13 | Results reported in sufficient detail? | NA |
| 14 | Do the results support the conclusions? | NA |

Appendix B. Note that this is not an exhaustive account of the instructions given; the manual provides guidance for circumstances under which “Yes,” “No,” or “Partially” should be indicated. Additionally, on the data extraction form, extra sub-questions were created for each item, which assisted the data extraction team in determining the score for each question in a systematic manner.

**Appendix C**

Gender, Sex, and Autism Terminology in Quantitative and Qualitative Studies

|  | ***n* of Studies** | **Percentage** |
| --- | --- | --- |
| **Reporting of participants' gender identities:** |  |  |
| Gender identities were fully reported | 53 | 63.00% |
| Only whether participants were trans or cis was reported | 4 | 4.76% |
| Only whether participants were a binary or non-binary gender was reported | 1 | 1.19% |
| Gender identities were not at all reported | 26 | 30.95% |
| **Term used to report current gender:** |  |  |
| Gender identity | 43 | 51.19% |
| Gender | 10 | 11.90% |
| Self-identified gender | 2 | 2.38% |
| Experienced gender | 1 | 1.19% |
| Current gender identity | 1 | 1.19% |
| Preferred gender designation | 1 | 1.19% |
| Primary reported gender | 1 | 1.19% |
| NA (gender was not reported) | 25 | 29.76% |
| **Types of gender options included:** |  |  |
| Binary options only (e.g., man, woman) | 8 | 9.52% |
| Binary and nonbinary options | 48 | 57.14% |
| NA (gender was not reported) | 28 | 33.33% |
| **Reporting of participants' SAB:** |  |  |
| SAB was reported | 70 | 83.33% |
| SAB was not reported | 14 | 16.67% |
| **Term used to report SAB** |  |  |
| Sex-based term (e.g., sex assigned at birth) | 53 | 63.10% |
| Gender-based term (e.g., assigned gender) | 15 | 17.86% |
| NA (SAB was not reported) | 16 | 19.05% |
| **Types of SAB options included:** |  |  |
| Male and female options only | 61 | 72.62% |
| Male, female, and intersex/other option | 7 | 8.33% |
| NA (SAB was not reported) | 16 | 19.05% |
| **Term for people diagnosed with autism** |  |  |
| People with autism/ASD | 45 | 53.57% |
| Autistic people | 39 | 46.43% |
| **Term for people identifying as gender-diverse** |  |  |
| People with gender dysphoria | 24 | 28.57% |
| Transgender AND gender nonconforming OR nonbinary OR gender-diverse people | 16 | 19.05% |
| Gender-diverse people | 13 | 15.48% |
| Transgender people | 11 | 13.10% |
| People with gender variance | 5 | 5.95% |
| Gender minority people | 4 | 4.76% |
| LGBTQ+ people | 3 | 3.57% |
| People identifying as "other" gender | 2 | 2.38% |
| Gender nonconforming people | 1 | 1.19% |
| People across the gender spectrum | 1 | 1.19% |
| Transsexual people | 1 | 1.19% |
| People with low gender self-concept | 1 | 1.19% |
| Nonbinary people | 1 | 1.19% |
| NA (no term used) | 1 | 1.19% |

**Appendix D**

Gender Diversity and Autism Measures in Quantitative Studies

**Table 1**

*Gender Diversity Measures Used in Quantitative Studies*

|  | ***n* of Studies** | **Percentage** |
| --- | --- | --- |
| **Methods of measuring gender diversity variables** |  |  |
| Categorical | 46 | 73.02% |
| Continuous | 10 | 15.87% |
| Continuous and categorical | 7 | 11.11% |
| **Measures of gender diversity-related variables** |  |  |
| ***Self-Report Measures*** |  |  |
| Self-report surveys | 22 | 34.92% |
| Gender Identity/Gender Dysphoria Questionnaire for Adolescents and Adults | 7 | 11.11% |
| Utrecht Gender Dysphoria Scale | 4 | 6.35% |
| Recalled Childhood Gender Identity/Gender Role Questionnaire | 3 | 4.76% |
| Implicit Associations Test | 2 | 3.17% |
| Wish to be of the Opposite Sex (Adult Self-Report) | 2 | 3.17% |
| Wish to be of the Opposite Sex (Youth Self-Report) | 2 | 3.17% |
| Collective Self-Esteem Scale | 1 | 1.59% |
| Gender Conformity/Nonconformity Scale | 1 | 1.59% |
| Gender Diversity Screening Questionnaire-Self | 1 | 1.59% |
| Gender Minority Stress and Resilience Measure | 1 | 1.59% |
| Gender Self-Report | 1 | 1.59% |
| Marquette Measure of Gender Conformity | 1 | 1.59% |
| Personal Attributes Questionnaire | 1 | 1.59% |
| Social Identification Scale | 1 | 1.59% |
| ***Parent-Report Measures*** |  |  |
| Wish to be of the Opposite Sex (Child Behavior Checklist) | 4 | 6.35% |
| Gender Identity Questionnaire for Children | 3 | 4.76% |
| Gender Diversity Screening Questionnaire-Parent | 1 | 1.59% |
| ***Clinician-Report Measures*** |  |  |
| DSM-5 interview for Gender Dysphoria | 9 | 14.29% |
| DSM-IV interview for Gender Identity Disorder | 2 | 3.17% |
| ICD-10 interview for Gender Identity Disorder | 1 | 1.59% |
| ***Other*** |  |  |
| Inferred gender diversity based on gender clinic referral | 12 | 19.05% |
| EMR review for DSM-5 Gender Dysphoria | 5 | 7.94% |
| EMR review for ICD-9-CM Gender Identity Disorder | 2 | 3.17% |

**Table 2**

*Autism Measures Used in Quantitative Studies*

|  | ***n* of Studies** | **Percentage** |
| --- | --- | --- |
| **Methods of measuring autism-related variables** |  |  |
| Categorical | 30 | 47.62% |
| Continuous and categorical | 20 | 31.75% |
| Continuous | 13 | 20.63% |
| **Measures of autism-related variables** |  |  |
| ***Self-Report Measures*** |  |  |
| Self-report survey | 18 | 28.57% |
| Autism Spectrum Quotient-50 | 14 | 22.22% |
| Autism Spectrum Quotient-28 | 4 | 6.35% |
| Ritvo Autism and Asperger Scale-14 | 3 | 4.76% |
| Autism Spectrum Quotient-10 | 2 | 3.17% |
| Broad Autism Phenotype Questionnaire | 2 | 3.17% |
| Camouflaging of Autistic Traits Questionnaire | 2 | 3.17% |
| Adult Social Behaviour Questionnaire | 1 | 1.59% |
| Social Responsiveness Scale-Adult | 1 | 1.59% |
| ***Parent-Report Measures*** |  |  |
| Social Responsiveness Survey-2 | 7 | 11.11% |
| Children's Social Behaviour Questionnaire | 3 | 4.76% |
| Parent-report survey | 3 | 4.76% |
| Social Communication Questionnaire | 3 | 4.76% |
| Autism Spectrum Quotient-Adolescent | 1 | 1.59% |
| Autism Spectrum Quotient-Child | 1 | 1.59% |
| CBCL 10-Item Autism Screener | 1 | 1.59% |
| ***Clinician-Report Measures*** |  |  |
| DSM-5 clinical interview for ASD | 4 | 6.35% |
| Autism Diagnostic Interview-Revised | 3 | 4.76% |
| Autism Diagnostic Observation Schedule-2 | 3 | 4.76% |
| ***Other*** |  |  |
| EMR review for ASD | 14 | 22.22% |
